# Dexmedetomidine alleviates olfactory cognitive dysfunction by promoting neurogenesis in the subventricular zone of hypoxic-ischemic neonatal rats

**DOI:** 10.3389/fphar.2022.983920

**Published:** 2022-08-19

**Authors:** Andi Chen, Xiaohui Chen, Jianhui Deng, Jianjie Wei, Haitao Qian, Yongxin Huang, Shuyan Wu, Fei Gao, Cansheng Gong, Yanling Liao, Xiaochun Zheng

**Affiliations:** ^1^ Department of Anesthesiology, Fujian Provincial Hospital, Shengli Clinical Medical College of Fujian Medical University, Fuzhou, China; ^2^ Fujian Provincial Key Laboratory of Emergency Medicine, Fujian Provincial Key Laboratory of Critical care Medicine, Fujian Provincial Co-Constructed Laboratory of “Belt and Road”, Fujian Emergency Medical Center, Fuzhou, China

**Keywords:** neonate, hypoxic-ischemic, dexmedetomidine, neurogenesis, subventricular zone, BDNF

## Abstract

**Background:** Hypoxic-ischemic brain damage (HIBD) is the main cause of neurological dysfunction in neonates. Olfactory cognitive function is important for feeding, the ability to detect hazardous situations and social relationships. However, only a few studies have investigated olfactory cognitive dysfunction in neonates with HIBD; furthermore, the specific mechanisms involved are yet to be elucidated. It has been reported that neurogenesis in the subventricular zone (SVZ) is linked to olfactory cognitive function. Recently, dexmedetomidine (DEX) has been shown to provide neuroprotection in neonates following HIBD. In the present study, we investigated whether DEX could improve olfactory cognitive dysfunction in neonatal rats following HIBD and attempted to determine the underlying mechanisms.

**Methods:** We induced HIBD in rats using the Rice–Vannucci model, and DEX (25 μg/kg, i.p.) was administered immediately after the induction of HIBD. Next, we used triphenyl tetrazolium chloride (TTC) staining and the Zea-longa score to assess the success of modelling. The levels of BDNF, TNF-α, IL-1β and IL-6 were determined by western blotting. Immunofluorescence staining was used to detect microglial activation and microglial M1/M2 polarization as well as to evaluate the extent of neurogenesis in the SVZ. To evaluate the olfactory cognitive function, the rats in each group were raised until post-natal days 28–35; then, we performed the buried food test and the olfactory memory test.

**Results:** Analysis showed that HIBD induced significant brain infarction, neurological deficits, and olfactory cognitive dysfunction. Furthermore, we found that DEX treatment significantly improved olfactory cognitive dysfunction in rat pups with HIBD. DEX treatment also increased the number of newly formed neuroblasts (BrdU/DCX) and neurons (BrdU/NeuN) in the SVZ by increasing the expression of BDNF in rat pups with HIBD. Furthermore, analysis showed that the neurogenic effects of DEX were possibly related to the inhibition of inflammation and the promotion of M1 to M2 conversion in the microglia.

**Conclusion:** Based on the present findings, DEX treatment could improve olfactory cognitive dysfunction in neonatal rats with HIBD by promoting neurogenesis in the SVZ and enhancing the expression of BDNF in the microglia. It was possible associated that DEX inhibited neuroinflammation and promoted M1 to M2 conversion in the microglia.

## 1 Introduction

Neonatal hypoxic-ischemic brain damage (HIBD) is the main factor responsible for neonatal mortality and subsequent disability in adulthood and is caused by perinatal asphyxia (Chavez-Valdez et al., 2021). Despite recent advances in perinatal medicine, the associated rate of disability of HIBD in neonates has increased year-by-year, although the mortality has improved significantly (Finder et al., 2020). According to the literature, 1 to 3 in every 1,000 newborns experience HIBD each year worldwide; 25% of those that survive moderate-to-severe HIBD will suffer from permanent neurological deficits (Ambalavanan et al., 2021). Neonatal HIBD often causes audiovisual impairment, cerebral palsy, cognitive dysfunction, memory difficulties and other neurological sequelae (Disdier and Stonestreet, 2020; Finder et al., 2020; Zhao et al., 2021). Nevertheless, only a few studies (Drobyshevsky et al., 2006; Juliano et al., 2015) have investigated olfactory cognitive dysfunction in neonates with HIBD; the specific mechanisms underlying this condition remain unclear.

Olfactory cognitive function is important for feeding, the ability to detect hazardous situations and social relationships (Doty and Kamath, 2014); these activities involve several distinct abilities, especially odor identification and memory (Chen B. et al., 2022). The first step in the formation of the sense of smell begins in the nose. Olfactory information is transmitted from the olfactory epithelium to the olfactory bulbs (OB); then, the OB transmits olfactory information to secondary and tertiary olfactory structures *via* neurons (Mori and Sakano, 2021). A number of factors can influence olfactory cognitive function, including genetic factors, physical activity, nutrition, gender, head trauma, exposure to viruses and medical treatment (Doty, 2017). Research has identified various degrees of olfactory cognitive dysfunction in multiple conditions of brain injury, such as traumatic brain injury (Siopi et al., 2012; Ciofalo et al., 2018; Powell et al., 2019), acute ischemic stroke (Okamoto et al., 2020) and sevoflurane-induced brain damage (Kostopanagiotou et al., 2011). These studies have shown that the loss of neurons in the olfactory bulb is a crucial cause of olfactory cognitive dysfunction. The importance of olfactory cognitive function lies in its significant influence in other social behaviors, including personality, sexual behavior and interpersonal communication (Sari et al., 2020). Once abnormalities in olfactory cognitive function occur, they may lead to severe psychiatric disorders, such as depression, schizophrenia, and olfactory cognitive dysfunction (Schinder and Gage, 2004; Apple et al., 2017). It has been previously confirmed that HIBD causes the extensive death of neurons (Yıldız et al., 2017; Chen et al., 2021); this is similar to the main mechanisms underlying olfactory cognitive dysfunction described above. Therefore, in the present study, we focused on whether HIBD could significantly impair olfactory cognitive function in neonates and searched for effective treatments that might offer a fresh perspective on the neurological deficit associated with neonatal HIBD.

Neurogenesis is the process by which neural stem cells proliferate and differentiate into directed progenitor cells by symmetrical and asymmetrical division. Subsequently, they progressively migrate to functional areas and undergo continuous plastic changes, eventually establishing synaptic connections with other neurons to produce neurological functionality (Li and Guo, 2021; Rizk et al., 2021). In the developing brain, neurogenesis persists in two neurogenic niches (Poiana et al., 2020; Cebrian-Silla et al., 2021; Du et al., 2021). In the sub-granular zone (SGZ) of the dentate gyrus, neurogenesis has been linked to both memory and learning (Okamoto et al., 2021). In the subventricular zone (SVZ), newly formed neurons can migrate to the OB and integrate into neural circuits; this process is important for olfactory recognition memory (Sha et al., 2021). Disruptions in SVZ neurogenesis may cause abnormalities in the neural circuitry, thus resulting in olfactory cognitive dysfunction and subsequent serious mental illnesses such as depression and schizophrenia (North et al., 2021). Previous research has shown that brain-derived neurotrophic factor (BDNF) plays an important role in neurogenesis (Vinberg et al., 2015); this factor is widely expressed in all types of brain cells, especially M2 microglia (Zhou et al., 2021). BDNF promotes neuronal growth, reduces the loss of neurons, and promotes neurogenesis (Xu et al., 2020), thus highlighting the importance of BDNF in the repair of neurological damage. Recent evidence suggests that the promotion of neurogenesis can improve narcotic-induced olfactory cognitive dysfunction (Sha et al., 2021). Therefore, it can be concluded that promoting SVZ neurogenesis by enhancing the expression of BDNF could be a promising strategy to improve the olfactory cognitive dysfunction in neonatal HIBD.

Dexmedetomidine (DEX) is a highly selective α-2 agonist of the adrenergic receptor and is used clinically as an analgesic, sedative and anxiolytic medication (Liu et al., 2018). Several researchers (Dong et al., 2020; Gao et al., 2020; Liaquat et al., 2021; Mei et al., 2021) have demonstrated that DEX exhibits neuroprotective effects for brain injury by promoting neurogenesis, anti-inflammatory effects, and by increasing intravascular calcium and reducing the levels of catecholamine. Although previous studies have shown that DEX exhibits neuroprotective effects against brain injury, the molecular mechanisms and signaling pathways that mediate these effects have yet to be elucidated. In view of this, we hypothesized that DEX treatment could improve olfactory cognitive dysfunction in neonatal rats with HIBD by promoting neurogenesis in the SVZ by enhancing the expression of BDNF.

In the present study, to validate our hypothesis, we investigated whether HIBD could induce severe olfactory cognitive dysfunction in neonatal rats and whether DEX could improve olfactory cognitive dysfunction in neonatal rats following HIBD by promoting neurogenesis by upregulating BDNF expression and its upstream mechanisms.

## 2 Materials and methods

### 2.1 Animals

We obtained nursing Sprague-Dawley (SD) rats and their offspring from Fujian Medical University. All animal experiments were carried out according to the Guide for the Care and Use of Laboratory Animals. Each neonatal rat was kept with its littermates and the nursing rat in a cage under controlled conditions of temperature (25 ± 2°C) and light (12 h of light/12 h of darkness). All animals had free access to water and food. A total of 268 neonatal rats were used for this study and randomly divided into different experimental groups.

### 2.2 Animal model and the experimental protocol

#### 2.2.1 Establishment of the hypoxic-ischemic brain damage model

Most of the published studies involving a neonatal HIBD model have used the Rice-Vannucci model (Rice et al., 1981). In the present study, our animal model was constructed as described previously (Rice et al., 1981). First, we used 3% isoflurane to anesthetize 7-day-old SD rats. Subsequently, in order to expose the left common carotid artery, a skin incision of 0.5 cm was incised in the middle of the neck; then, we performed blunt dissection. Next, a 5–0 surgical suture was used to ligate the left common carotid artery. After suturing the skin, the neonatal rats were placed back to their cage with their dam for approximately 1 h to regain consciousness. Finally, the rat pups were placed into a hypoxic chamber (MCO 18M; Sanyo Biomedical Electrical Co., Ltd., Tokyo, Japan) filled with 8% oxygen and 92% nitrogen for 2 h at 37°C. Simultaneously, an atomizer was used to maintain the humidity at 50%–70% in the chamber.

#### 2.2.2 Experimental protocol 1

First, we assessed whether HIBD leads to severe olfactory cognitive dysfunction in neonatal rats. The rat pups were assigned to two groups: 1) Sham-operated rats (the sham group in which the rats were anesthetized and the left common carotid artery was exposed without ligation or hypoxia.), and 2) an experimental group in which rat pups were exposed to hypoxia-ischemia (the HI group) (*n* = 10 per group). The Zea-longa score was determined and triphenyl tetrazolium chloride (TTC) staining was performed within 2 days of hypoxia-ischemia treatment (HI treatment) (Rice et al., 1981) to assess whether the HIBD model had been successfully established. The buried food test and the olfactory memory test were performed 21–28 days after HI treatment to assess whether HIBD led to olfactory cognitive dysfunction in the neonatal rats.

#### 2.2.3 Experimental protocol 2

**FIGURE 1:**
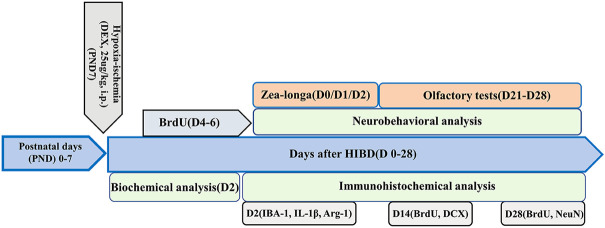
Flowchart of the experimental design deployed in the present study. Hypoxic-ischemic brain damage was induced in 7-day-old (PND 7) Sprague-Dawley rats. The rats were divided into three groups: a sham-operated group, a hypoxic-ischemic (HI) group and a post-HI DEX (25 μg/kg, i.p.) intervention group (HI + DEX). A proportion of the animals in these three groups underwent Zea-Longa score analysis, biochemical analysis, and immunofluorescence analysis on day 2 after HI (D2). Some of the rats in these three groups were tested for neurogenesis by immunofluorescence analysis and received BrdU injections twice daily for 3 consecutive days starting on day 4 (D4); These animals were perfused trans-cardially with 4% paraformaldehyde 14 and 28 days after the insult. The remaining animals received olfactory tests on D21–D28.

Next, we investigated whether DEX could improve olfactory cognitive dysfunction in neonatal rats with HIBD and attempted to identify the underlying mechanisms. Rat pups were divided into three groups: 1) a Sham group with vehicle (saline) (the Sham group), 2) a group in which rat pups underwent hypoxia-ischemia with a vehicle (saline) (the HI group) and 3) a group in which rat pups underwent hypoxia-ischemia with DEX (25 μg/kg, Sigma-Aldrich, St. Louis, MO, United States) which was administered intraperitoneally immediately after exposure to hypoxia-ischemia. The administration dose and route of DEX were selected based on our previous neuroprotective studies (Chen et al., 2021). The sham group and HI group received the same volume of saline as the vehicle group. The buried food test and the olfactory memory test were performed 21–28 days after HI treatment to assess whether DEX could improve olfactory cognitive dysfunction in neonatal rats with HIBD. Western blotting and immunofluorescent analyses were performed to identify the underlying mechanisms. The experimental design of this study is summarized in Figure 1.

### 2.3 Bromodeoxyuridine labeling

Endogenous cell proliferation was detected by the incorporation of 5-bromo-2-deoxyuridine (BrdU), an indicator of mitosis. BrdU (B5002, Sigma) was administered intraperitoneally (50 mg/kg per injection, in sterile 0.9% NaCl plus 0.007 N NaOH). Previous research showed that cell proliferation was most intensive 4–6 days after HI treatment (Jaworska et al., 2019). Therefore, to investigate the extent of neurogenesis in the SVZ, rat pups received BrdU injections twice daily (12 h apart) 4–6 days after HI treatment. Animals were sacrificed 14 and 28 days after HI treatment in this group.

### 2.4 Zea-longa score

The Zea-longa score is a classic neurological assessment method (Longa et al., 1989). To assess the successful establishment of the HIBD model, the Zea-longa score was determined at 0, 6, 12, 24 and 48 h after HI treatment. The scoring metrics were defined as previously (Longa et al., 1989), as follows: 0 points, no obvious neurological deficits; 1 point, flexion of the contralateral torso and limbs when held by the tail; 2 points, turning to the affected side when held by the tail; 3 points, leaning towards the affected side and 4 points, no spontaneous locomotion.

### 2.5 Triphenyl tetrazolium chloride staining

To assess the successful establishment of the HIBD model from the pathological point-of-view, we performed TTC staining to detect the infarct volume of the ipsilateral injury hemisphere in neonatal rats with HIBD. Two days after HI treatment, rat pups were sacrificed under deep anesthesia; then, the brains were removed immediately. Brain tissues were frozen at −20°C for 20 min, and then sectioned into 2 mm coronal sections. Afterwards, the brain sections were immersed in 4% TTC solution (17779, Sigma-Aldrich) in the dark. Finally, the sections were rinsed in phosphate buffered saline (PBS, P3813, Sigma-Aldrich) and fixed in 4% formaldehyde.

### 2.6 Buried food test

In this study, the buried food test was performed to evaluate the ability of neonatal rats to detect odors. We used a 35 cm × 20 cm × 15 cm cage as the experimental device; this was placed with 5 cm padding. Afterwards, feed was buried in a randomly selected location in the testing cage 1.5 cm below the surface. All rat pups were tested between 10:00 a.m. and 1:00 p.m. prior to the actual test, rat pups were placed in the experimental device and allowed to acclimatize to the environment. They were then allowed to find the hidden feed for 5 min on three consecutive training days. In the formal experiment, the rat pups were fasted for 12 h; then, the rat pups were allowed to freely explore the experimental cage and find the hidden feed. Rat pups failed the search test if they could not find the feed within 5 min; in these cases, we recorded the search time as 5 min. Feed was buried in a randomly selected location each day and the final results were expressed as the mean of four consecutive days (*n* = 10 animals/group).

### 2.7 Olfactory memory test

In the olfactory memory test, animals are tested for their ability to recognize and remember smells. We used a 55 cm × 30 cm × 25 cm cage as the experimental device; this was separated into two compartments of the same size by two opaque partitions with a gap of 5 cm at the bottom of each partition. Filter papers impregnated with 10 μl of cinnamon essential oil and 10 μl of ginger essential oil were placed in the left and right compartments, respectively. Prior to any test, all animals were fasted for 12 h. Prior to the actual test, the feed was placed in the compartment with ginger essential oil; subsequently, rat pups were placed in the experimental device and allowed to freely explore for 5 min for three consecutive training days. We used alcohol to clean the device during the interval between each exploration. In the formal experiment, feed was removed from the compartment with ginger essential oil. We recorded how much time rat pups spent exploring each compartment within a 5 min period (*n* = 10 animals/group).

### 2.8 Tissue preparation and immunofluorescence staining

Animals were deeply anesthetized with 3% isoflurane (RWD, Shenzhen) and then trans-cardially perfused with 0.9% sodium followed by 4% paraformaldehyde. Afterwards, the brains were removed immediately and fixed overnight in 4% paraformaldehyde at 4°C. Then, the brain tissues were embedded in paraffin and sectioned in the coronal plane at a thickness of 3.0–3.5 μm. Finally, sections were deparaffinized. Sections were incubated with the following antibodies (source, catalog number, and final dilution): rabbit polyclonal anti-doublecortin (DCX; Cell signaling, 4694, 1:200), mouse monoclonal anti-NeuN (Millipore, MAB377, 1:200), sheep polyclonal anti-BrdU (Abcam, ab 1893, 1:500), rabbit monoclonal anti-BDNF (Abcam, ab108319, 1:200), mouse monoclonal anti-Iba1 (Abcam, EPR16589, 1:100), rabbit polyclonal anti-IL-1β (Santa Cruz, sc-7884, 1:250) and goat polyclonal anti-Arg-1 (arginase-1) (Santa Cruz, sc-18354, 1:250).

To determine the extent of neurogenesis, we conducted double immunofluorescence staining to identify new neuroblasts and neurons in the ipsilateral injury hemisphere of the brain sections at various stages of maturation. The selected sections were first deparaffinized and hydrated. These slices were baked in a 60°C oven for 20 min and then placed in xylene for 10 min. Subsequently, the sections were placed in fresh xylene for another 10 min. Afterwards, the sections were placed into anhydrous ethanol, 95% ethanol and 75% ethanol for 5 min, respectively. After dewaxing and hydrating, the coronal brain sections were used for BrdU/DCX and BrdU/NeuN double-label immunofluorescence staining to detect new neuroblasts and neurons, respectively. The sections were treated with 2 N HCL for 30 min at 37°C for DNA denaturation and with 0.1 M borate buffer (pH 8.5) for neutralization. Then, the sections were blocked by incubation in 5% fetal bovine serum for 3 min, washed three times in PBS and then incubated overnight with primary antibodies at 4°C. Subsequently, double staining was performed on immunolabeled tissue slices by incubation with two types of secondary antibodies for 2 h at room temperature. Finally, the sections were counterstained with DAPI.

To detect the levels of BDNF expression and whether BDNF was expressed on the microglia, we performed double immunofluorescence on day 2 after HI. The processes used for dewaxing, hydration and immunofluorescence staining were described in the previous section. Then, we labeled microglia and BDNF in the ipsilateral injury hemisphere of the brain sections with anti-Iba1 and anti-BDNF, respectively.

Next, we investigated the activation of microglia and the polarization of microglia on day 2 after HI treatment. The processes used for dewaxing, hydration and immunofluorescence staining were described in the previous section. Then, we labeled microglia and the proportions of M1 and M2 fractions in the ipsilateral injury hemisphere of brain sections with anti-Iba1, anti-IL-1β and anti-Arg-1, respectively. The positive cells were manually counted by a skilled laboratory technician using the cell counter function of imageJ 1.4 software under a laser scanning confocal microscope (Fluoview 1000, Olympus). Regarding the methods for assessing the number of M1 (IBA-1^+^/IL-1β^+^) and M2 (IBA-1^+^/Arg-1^+^) microglia, one slice was randomly selected from each animal and used to calculate the number of IBA-1^+^, IBA-1^+^/IL-1β^+^ and IBA-1^+^/Arg-1^+^ cells as well as the proportion of IBA-1^+^/IL-1β^+^ cells in IBA-1^+^ cells and the proportion of IBA-1^+^/Arg-1^+^ cells in IBA-1^+^ cells.

### 2.9 Western blot analysis

Western blotting was carried out as described previously (Chen X. et al., 2022). Tissues from the left brain hemisphere were collected on day 2 after HI treatment and homogenized using lysis buffer supplemented with protease and phosphatase inhibitors on ice. Equal amounts of protein (40 μg/well) were then separated by SDS-PAGE and transferred to polyvinylidene fluoride membranes (EMD Millipore). Next, 5% non-fat milk was diluted in Tris-buffered saline with Trion X (TBST) for 1.5 h to block the membranes. The membranes were then incubated with primary antibodies overnight at 4°C against the following proteins: rabbit monoclonal anti-BDNF (Abcam, ab108319, 1:1000), rabbit monoclonal anti- IL-1β (Abcam, ab254360, 1:1000), rabbit polyclonal anti-TNF-α (Abcam, ab6671, 1:1000) and rabbit monoclonal anti- IL-6 (Abcam, EPR21711, 1:1000). Afterwards, the membranes were incubated with the corresponding secondary antibodies (Abcam, ab205718, 1:20000) at room temperature for 2 h. Protein bands were visualized and photographed with ECL substrate kits (Abcam, ab133406) and a GE Amersham Imager 600. Finally, we used Image J to perform densitometric analysis of band intensity. By normalizing to β-actin, we obtained relative protein expression levels.

### 2.10 Statistical analysis

Data are presented as mean ± standard deviation and analyzed using SPSS 22.0 or GraphPad Prism 6.0 software. The student’s t-test was performed to compared data between two groups and one-way analysis of variance (ANOVA) was used to compare differences between three groups. *p* < 0.05 was defined as statistically significant.

### 2.11 Ethical statement

The Animal Care and Use Committee at Fujian Medical University (Fuzhou, China) approved this research (No: IACUC FJMU 2022-0463). During the animal experiments, we followed the National Research Council’s Guide for the Care and Use of Laboratory Animals. All efforts were aimed at reducing suffering and the numbers of experimental animals utilized.

## 3 Results

### 3.1 Dexmedetomidine alleviated olfactory cognitive dysfunction in neonatal rats caused by hypoxic-ischemic brain damage

**FIGURE 2:**
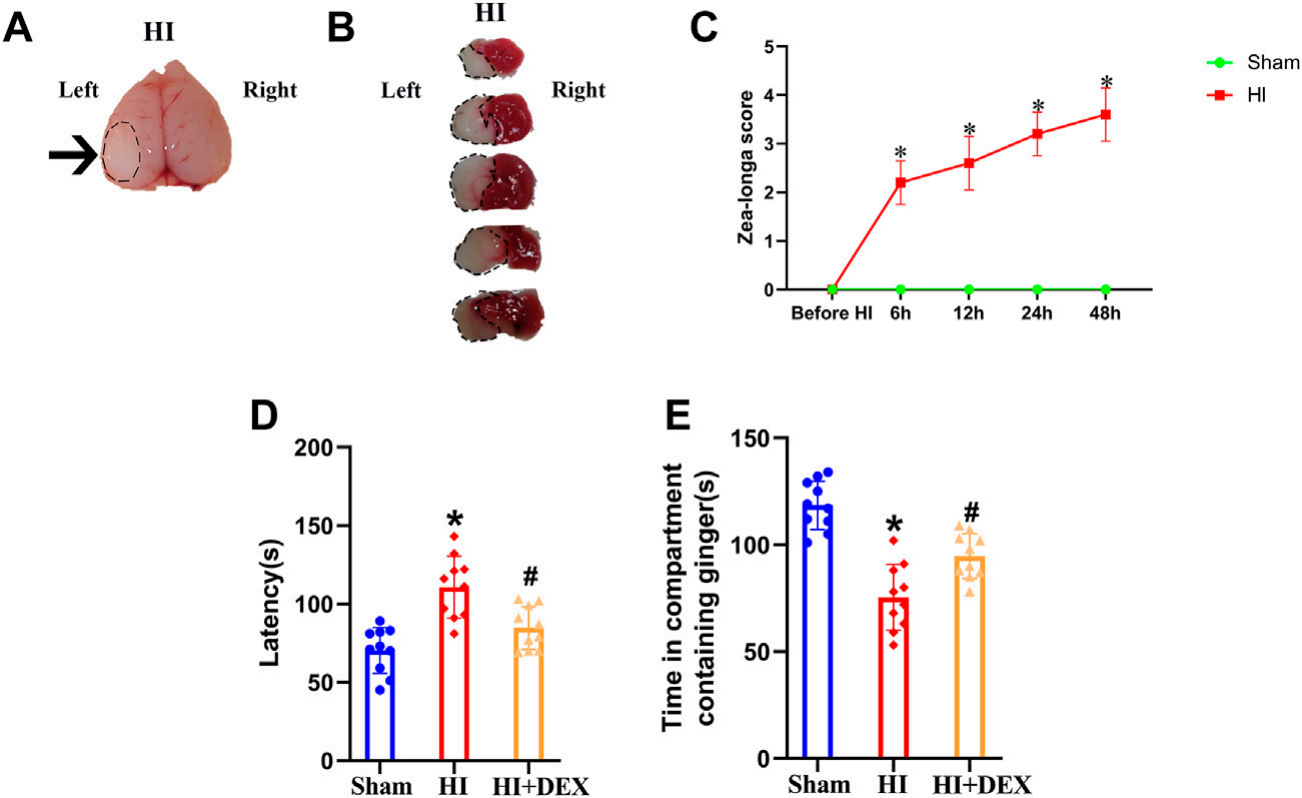
DEX alleviated long-term olfactory cognitive dysfunction in neonatal rats caused by HIBD. **(A)** Brain imaging in the HI group; the black arrow represents the white cerebral infarct focus of the ipsilateral injured hemisphere. **(B)** TTC staining of the brain from rats in the HI group. The black circular dotted lines depict the infarction zone. **(C)** Line chart showing Zea-longa score in the sham and HI groups before HI, and at 6, 12, 24 and 48 h post-insult. Data indicate that the scores in the HI group were significantly higher than those in the sham group. The buried food test **(D)** showed that the latency to find hidden feed within 5 min in the HI group was significantly longer than that in the sham group. The longer latency to find hidden feed caused by HIBD was partially reversed by DEX. The olfactory memory test **(E)** showed that the time that rats spent in the compartment containing ginger essential oil was significantly decreased in the HI group when compared with the sham group, but the olfactory memory impairment induced by HIBD was partially reversed by DEX. HIBD, Hypoxic-ischemic brain damage; HI, Hypoxia-ischemia; TTC, Triphenyl tetrazolium chloride; DEX, Dexmedetomidine; h, hour (s); min, minute (s). Data are expressed as the mean ± SD (*n* = 10 per group). *p < 0.05 vs. the sham group.

In this study, we first used TTC staining and the Zea-longa score to determine whether the HIBD model in 7-day-old rat pups had been successfully established. There was a white area in the ipsilateral injured hemisphere in the brain tissue of rats exposed to HI (Figure 2A), thus indicating the presence of HIBD-induced cerebral infarcts in the neonatal rats. Next, we used TTC staining to verify the pathological condition of the rat pups; the results were similar to the previous outcome in that there were obvious areas of infarction in the ipsilateral injured hemisphere of HI rats (Figure 2B). The Zea-longa scores of HI rats were significantly higher than those in the sham group at 0, 6, 12, 24 and 48 h (Figure 2C). These results indicated that our HIBD rat model had been successfully established. Based on this, we next investigated whether HIBD induced severe olfactory cognitive dysfunction in neonatal rats and whether DEX alleviated olfactory cognitive dysfunction in neonatal rats caused by HIBD. In the buried food test, rats in the HI group had a significantly longer latency than rats in the sham group. However, the longer latency to find hidden feed caused by HIBD was significantly reversed by DEX treatment (Figure 2D). In the olfactory memory test, rats in the HI group spent significantly less time in the compartment containing ginger essential oil than rats in the sham group, but rats in the HI + DEX group spent significantly more time in the compartment containing ginger essential oil than rats in the HI group (Figure 2E). Collectively, these results showed that HIBD induced severe olfactory cognitive dysfunction in neonatal rats and that DEX treatment alleviated olfactory cognitive dysfunction in neonatal rats with HIBD.

### 3.2 Dexmedetomidine promoted neurogenesis in the subventricular zone of hypoxic-ischemic neonatal rats

**FIGURE 3:**
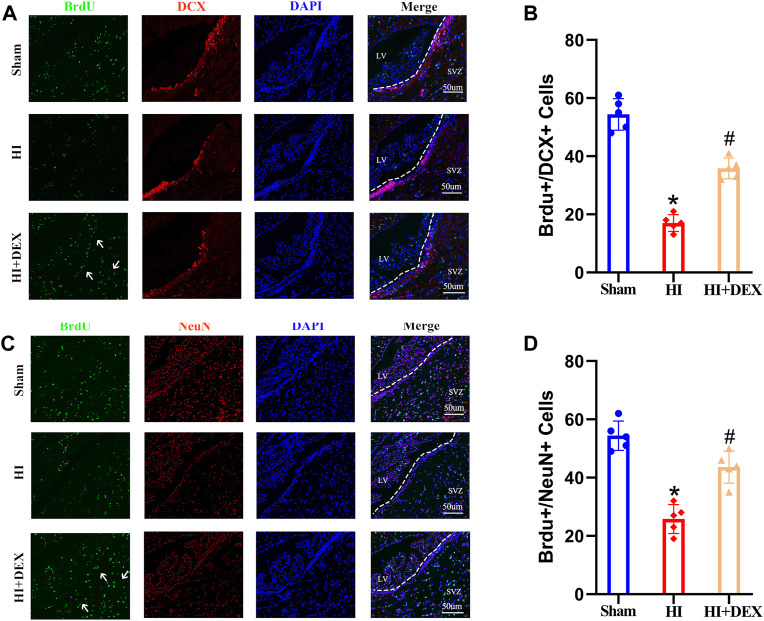
DEX promoted neurogenesis in the SVZ after HIBD. Neonatal HIBD was induced in rats on PND7. DEX (25 μg/kg) was administered directly by intraperitoneal injection after the onset of HIBD. BrdU was administered daily for three consecutive days starting on day 4 (D4). Rats in the sham group, HI group and HI + DEX group were trans-cardially perfused with paraformaldehyde and brain sections were taken on D14 and D28. The brain sections on D14 were double labeled with anti-BrdU (green) and anti-DCX (red) antibodies to detect newly formed neuroblasts. The brain sections on D28 were double labeled with anti-BrdU (green) and anti-NeuN (red) antibodies to detect newly formed neurons. **(A,B)** Representative images were captured using a laser scanning confocal microscope (magnification ×400, scale bar: 50 μm). The white arrows highlight a significant increase in the number of positive cells. **(C,D)** Statistical analysis of double-positive cells showed that the newly formed neuroblasts and newly formed neurons were significantly reduced in the SVZ of the ipsilateral injured hemisphere in neonatal rats caused by HIBD. This phenomenon was significantly reversed by DEX. DEX, Dexmedetomidine; SVZ, Subventricular zone; LV, Lateral ventricle; HIBD, hypoxic-ischemic brain damage; PND7, Postnatal day 7; D4, D14, D28: 4, 14, 28 Day after HIBD; HI, hypoxic-ischemia; The data are expressed as the mean ± SD (*n* = 5 per group). *p < 0.05 vs. the sham group; ^*#*^ *p <* 0.05 vs. the HI group.

Next, we investigated whether the improvement of olfactory cognitive impairment in neonatal rats with HIBD by DEX was related to its ability to promote neurogenesis in the SVZ. To detect neural stem cells that had differentiated into neural cells, we performed double labeling assays using BrdU with DCX, a marker of neuroblasts, and BrdU with NeuN, a marker of neurons. IF revealed that the number of new neuroblasts s in the SVZ was significantly lower in the HI group than that in the sham group, but exposure to DEX partially reversed this phenomenon (Figures 3A,B). A similar phenomenon was observed in the next set of IF results; the number of newly formed neurons was significantly reduced in the SVZ of neonatal rats with HIBD. Furthermore, the number of newly formed neurons was significantly higher in the SVZ of neonatal HIBD rats that had been treated with DEX (Figures 3C,D). Collectively, these results indicated that HIBD impaired olfactory cognitive function by disrupting the neurogenesis of SVZ in neonatal rats; in addition, DEX alleviated the olfactory cognitive dysfunction of hypoxic-ischemic neonatal rats by promoting neurogenesis in the SVZ.

### 3.3 Dexmedetomidine enhanced the expression of brain-derived neurotrophic factor in hypoxic-ischemic neonatal rats

**FIGURE 4:**
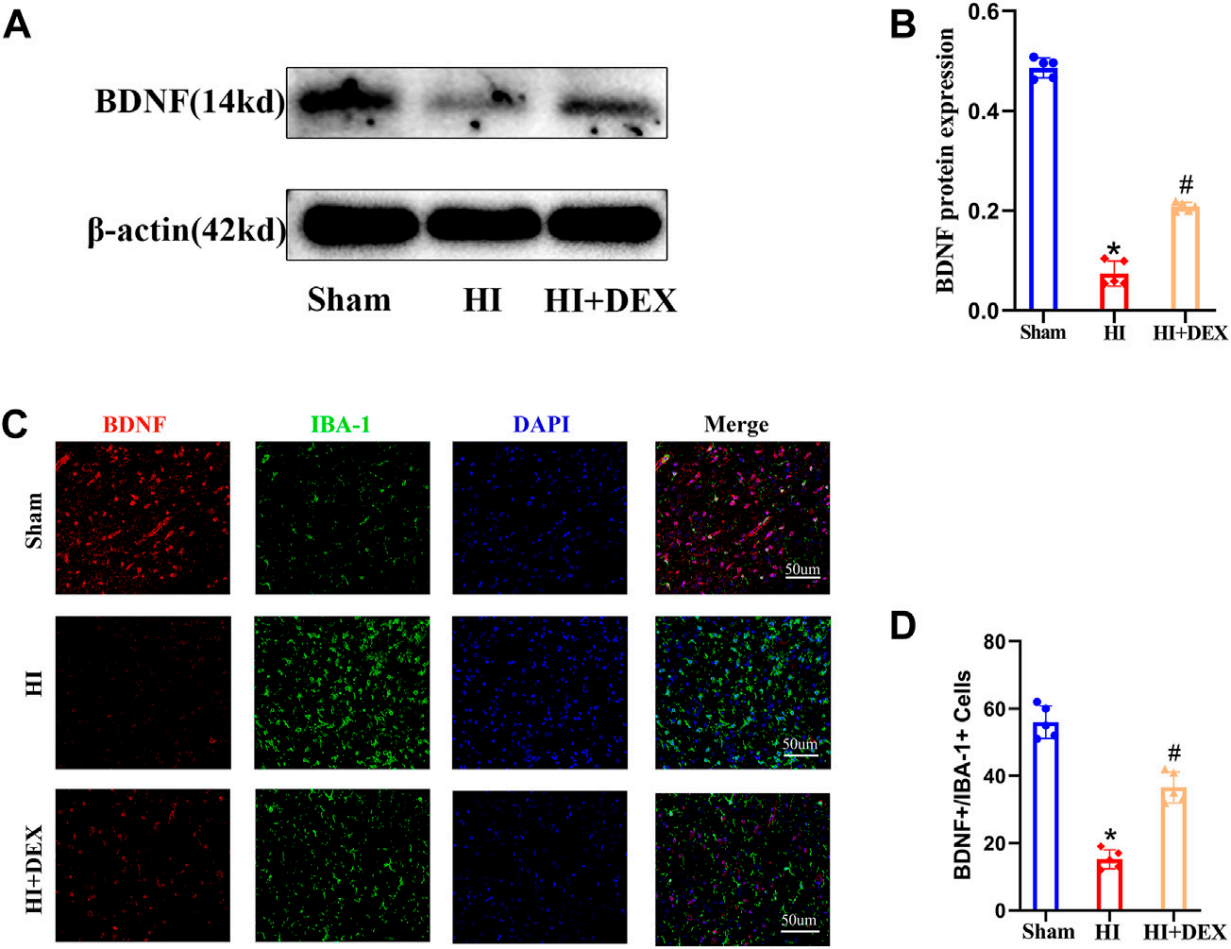
DEX enhanced the expression of BDNF in the ipsilateral injured hemisphere of neonatal rats with HIBD. Representative western blot image and quantitative analysis of the western blot (bar chart) illustrating the expression levels of BDNF in the ipsilateral injured hemisphere on D2 **(A,B)**. Representative IF image (magnification ×400, scale bar: 50 μm) and semi-quantitative analysis of the number of double-positive cells (BDNF^+^ and IBA^+^, a marker of microglia) showed that BDNF was expressed in the microglia and its expression on D2 **(C,D)**. IF image showing that BDNF was expressed in the microglia. Western blotting and IF results also showed that HIBD induced a reduction in BDNF expression and the activation of microglia; this phenomenon was partially reversed by DEX. DEX, Dexmedetomidine; BDNF, Brain derived neurotrophic factor; HIBD, hypoxic-ischemic brain damage; D2, 2 days after HIBD; IF, Immunofluorescence. The data are expressed as the mean ± SD (*n* = 5 per group). *p < 0.05 vs. the sham group; ^*#*^ *p <* 0.05 vs. the HI group.

**FIGURE 5:**
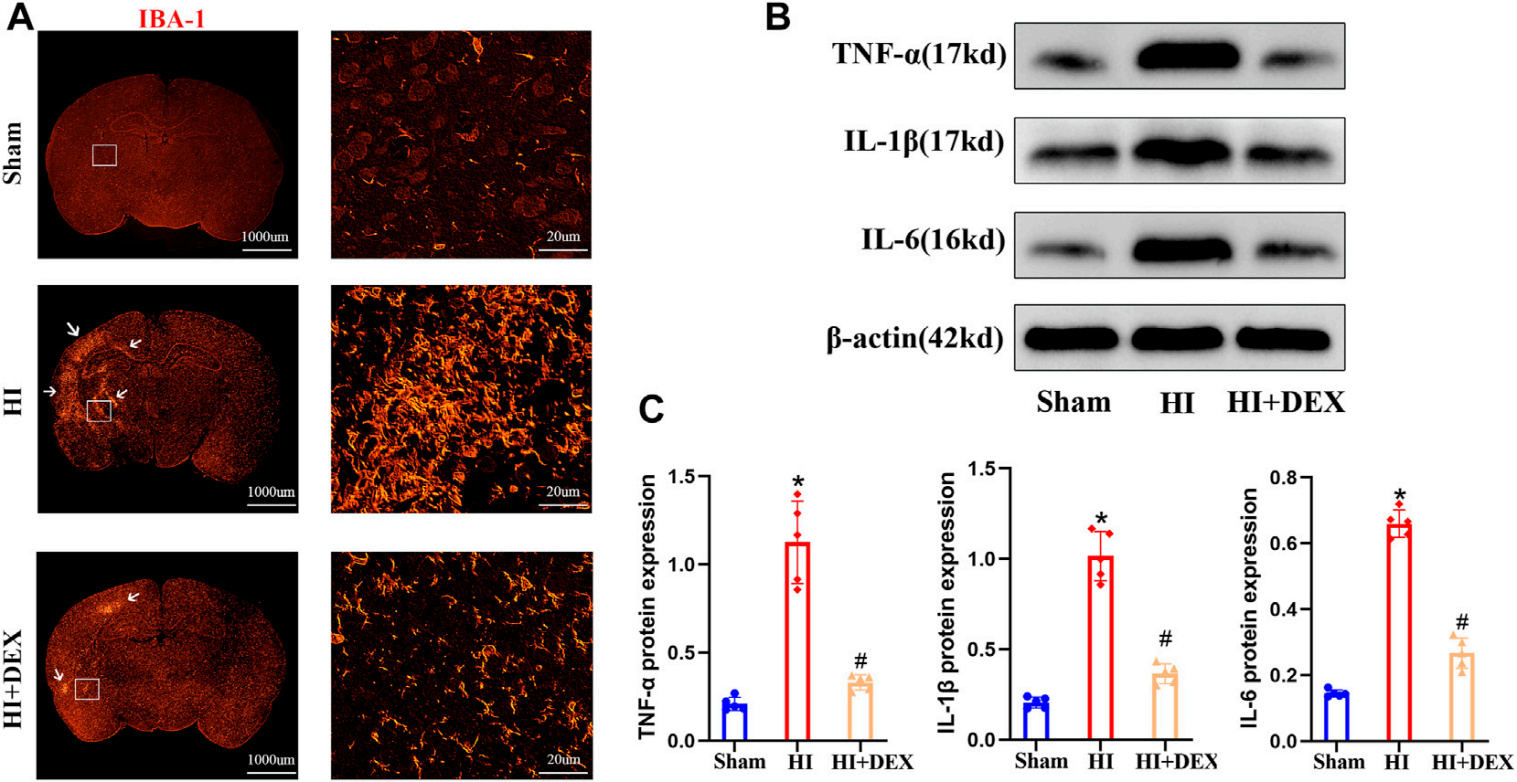
DEX reduced inflammatory factor protein expression and inhibited microglial activation in neonatal rats with HIBD. **(A)** Representative IF image (magnification ×1, scale bar: 1000 μm; magnification ×800, scale bar: 20 μm) showing the levels of microglial activation in the sham, HI and HI + DEX groups on D2. **(B–C)** Representative western blot image and quantitative analysis of the western blot (bar chart) illustrating the protein levels of crucial inflammatory factors, including TNF-α, IL-1β and IL-6, on D2. DEX, Dexmedetomidine; HIBD, Hypoxic-ischemic brain damage; IF, Immunofluorescence; HI, Hypoxic-ischemia; D2, 2 days after HIBD. Data are expressed as the mean ± SD (*n* = 5 per group). *p < 0.05 vs. the sham group; ^*#*^ *p <* 0.05 vs. the HI group.

BDNF is known to play a key role in neurogenesis, the differentiation of neural stem cells and neuronal survival (Lucini et al., 2018). To investigate whether DEX promoted neurogenesis in the SVZ by enhancing the expression of BDNF and whether BDNF was expressed by microglia, we used western blotting and IF to determine the levels of BDNF expression. Analysis showed that the levels of BDNF protein expression were significantly reduced in the HI group when compared with the sham group; however, DEX treatment significantly increased the levels of BDNF protein expression in hypoxic-ischemic neonatal rats (Figures 4A,B). Semi-quantitative immunofluorescent analyses showed the same results as the western blots. As shown in Figure 5C, BDNF was clearly colocalized with microglia (IBA-1, a marker of microglia). This IF result also showed that HIBD induced a consistently low level of BDNF-positive microglia in neonatal rats; furthermore, exposure to DEX reversed this mechanism of HIBD injury (Figures 4C,D). Collectively, these data indicated that DEX promoted neurogenesis in the SVZ by enhancing the expression of BDNF.

### 3.4 Dexmedetomidine reduced the levels of inflammatory factor protein expression and inhibited microglia activation in the hypoxic-ischemic neonatal rats

Based on the fact that BDNF is widely expressed in all forms of brain cells, including neurons (Zhou et al., 2021), and the anti-inflammatory effect of DEX (Mei et al., 2021), we hypothesized that DEX would significantly enhance the expression of BDNF by promoting cell survival *via* an anti-inflammatory effect. To confirm our hypotheses, we evaluated the effects of DEX on the activation of microglia and the expression levels of TNF-α, IL-1β and IL-6 in neonatal rats on day 2. IF analysis and western blotting showed that substantial microglial activation was detected in neonatal rats from the HI group when compared with the sham group. Moreover, exposure to DEX significantly reduced microglial activation when compared with HI rats (Figure 5A). As shown in Figure 5B, HIBD significantly increased the expression levels of TNF-α, IL-1 and IL-6 proteins in neonatal rats. In addition, DEX treatment significantly reduced the levels of TNF-α, IL-1β and IL-6 protein expression when compared with rats from the HI group (Figures 5B,C). Collectively, these data suggested that the one of the potential mechanisms responsible for the neuroprotective effects of DEX was anti-inflammatory effects.

### 3.5 Dexmedetomidine promoted the polarization of M1 to M2 microglia in hypoxic-ischemic neonatal rats

**FIGURE 6:**
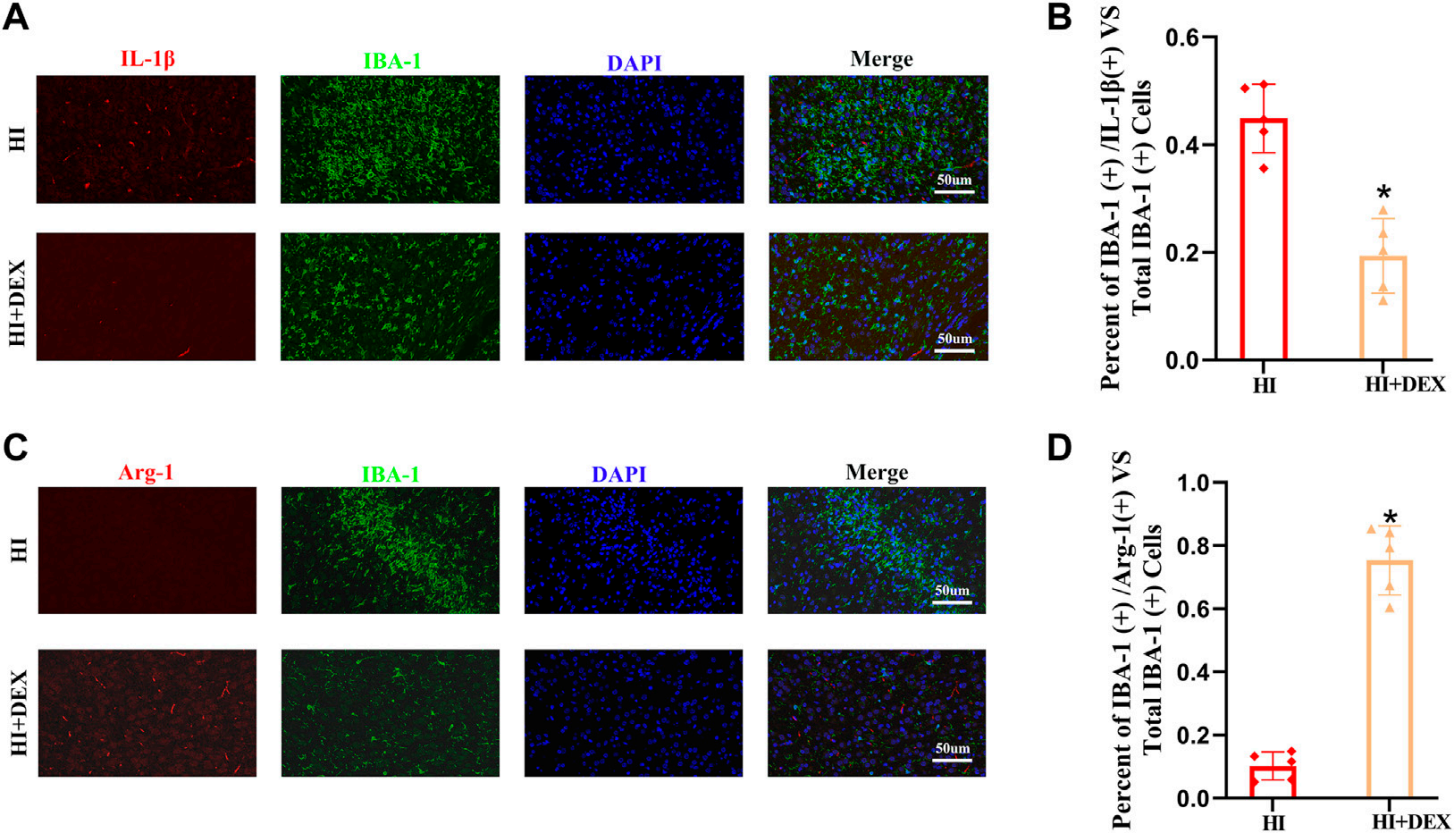
DEX promoted the polarization of microglia from the M1 to M2-like phenotype in neonatal rats with HIBD. Brain sections from the ipsilateral hemisphere on D2 were stained for anti-IBA-1 antibody (green) and **(A)** for anti-IL-1β, a marker for the M1 phenotype (red); and **(C)** for anti-Arg-1, a marker for the M2 phenotype (red). **(A–D)** Representative IF images (magnification ×400, scale bar: 50 μm) and semi-quantitative analysis (bar chart) of the ratios of IBA-1^+^\ IL-1β^+^ vs*.* total IBA-1^+^ and IBA-1^+^\ Arg-1^+^ vs*.* total IBA-1^+^ showing the status of microglia polarization. DEX, Dexmedetomidine; HIBD, Hypoxic-ischemic brain damage; D2, 2 days after HIBD. Data are expressed as the mean ± SD (*n* = 5 per group). *p < 0.05 vs. the HI group.

A previous study reported high expression levels of BDNF in M2 microglia (Bagheri et al., 2021). Therefore, we hypothesized that DEX would significantly enhance the expression of BDNF by promoting the polarization of M1 to M2 microglia. To test our hypotheses, we performed a double labeling IF assay using IBA-1 with IL-1β, a marker of M1 microglia, and IBA-1 with Arg-1, a marker of M2 microglia, to compare the microglia sub-types between the HI group and the HI + DEX group. We found that the number of M1 microglia in the HI + DEX group was significantly lower than that in the HI group (Figures 6A,B) and that the number of M2 microglia in the HI + DEX group was significantly higher than that in the HI group (Figures 6C,D). In summary, our results indicated that DEX enhanced the expression of BDNF by promoting the polarization of M1 to M2 microglia.

## 4 Discussion

In the present study, we demonstrated that HIBD induced severe olfactory cognitive dysfunction in neonatal rats by disrupting neurogenesis in the SVZ. Furthermore, DEX alleviated olfactory cognitive dysfunction in neonatal rats caused by HIBD by promoting neurogenesis in the SVZ and enhancing the expression of BDNF in the microglia. Analysis showed that the neurogenic effects of DEX were possibly related to the inhibition of inflammation and the promotion of M1 to M2 conversion in the microglia.

A number of studies have demonstrated that olfactory cognitive impairment can cause serious mental illness and act as a precursor to other serious brain disorders, including cerebral palsy and cerebral spasms (Apple et al., 2017; Yahiaoui-Doktor et al., 2019). In addition, a previous study reported that olfaction is crucial to human development from birth through adolescence and beyond and that the impairment of olfaction may compromise neonatal viability and affect long-term neural development (Schaal et al., 2020). However, studies involving the neurological sequelae of neonatal HIBD have failed to target olfactory cognitive dysfunction. A large number of molecules contribute to neonatal HIBD in all types of brain cells; these molecules can be divided into three groups (Millar et al., 2017): 1) excitotoxic, 2) oxidative and 3) inflammatory. The damage caused by these factors is extensive and irreversible, ultimately resulting in cell death. We hypothesized that HIBD may also impair olfactory cognitive function by disrupting neurogenesis of the SVZ, thus causing damage to neurons in the olfactory bulb in a manner dependent on the mechanism of injury. In our study, we used the buried food test and olfactory memory test to test the ability of neonatal rats to recognize and remember the smell of neonatal hypoxic-ischemic rats. Our results showed that HIBD caused severe olfactory cognitive dysfunction in neonatal rats. Based on this information, we next searched for effective treatments for HIBD-induced olfactory cognitive dysfunction and explored the mechanisms involved.

DEX is a selective alpha-2 adrenoreceptor agonist with superior analgesic efficacy and is commonly used in the pediatric outpatient clinic (Chen et al., 2019). In Europe, DEX is used for pediatric sedation; this application is supported by numerous clinical studies of pharmacological activity in children (Zhang et al., 2019). In recent years, DEX has attracted significant attention from researchers and doctors with regards to the treatment of neonates with HIBD. This is because DEX may offer neuroprotective effects on neurogenesis and neuronal plasticity. Therefore, this study selected DEX as a treatment to improve HIBD-induced olfactory cognitive dysfunction in a neonatal rat model. Using the buried food test and the olfactory memory test, we successfully confirmed that DEX improved HIBD-induced olfactory cognitive dysfunction, thus demonstrating that our hypothesis was correct.

Neurogenesis is the key process for neural development from embryonic to adult stages (Fan et al., 2012). Postnatal neurogenesis has recently been demonstrated to play a crucial role in the repair of nerve injury (Sha et al., 2021). In the SVZ, newly formed neurons migrate to the olfactory bulb and play an important role in olfactory cognitive function. Once neural disruption occurs in the SVZ, it has a significant impact on olfactory cognitive function. Previous studies have found that HIBD disrupted neurogenesis in the brain (Jaworska et al., 2019). Our immunofluorescence results also revealed that HIBD induced a significant reduction in newly formed neuroblasts and newly formed neurons in the SVZ. In addition, we found that DEX treatment promoted neurogenesis in the SVZ of neonatal rats following HIBD. Based on these findings, we hypothesized that the mechanisms underlying both HIBD-induced olfactory dysfunction and the ameliorative effect of DEX are related to neurogenesis in the SVZ. Therefore, we next investigated the upstream mechanisms of DEX in the neurogenesis of the SVZ in neonatal hypoxic-ischemic rats.

BDNF belongs to the neurotrophin family of growth factors (Xue et al., 2021) and is expressed at high levels in the developing brain; this protein has been proven to promote neurogenesis. Recently, a number of studies have reported that BDNF plays a crucial role in neurogenesis (Jaworska et al., 2019; Okamoto et al., 2021). The binding of BDNF to TrkB, a neurotrophin receptor, activates various intracellular signaling pathways, including the ERK, CREB and phosphoinositide 3-kinase pathways. Interestingly, ERK and CREB both play important roles during neurogenesis, brain development and neuronal proliferation (Degos et al., 2013). Previous research (Vilar and Mira, 2016) has indicated that BDNF/TrkB signaling pathway can promote the differentiation of neural stem cells into neurons and is involved in adult neurogenesis. Therefore, we focused our research on BDNF. Concurred with previous research (Jaworska et al., 2019; Xue et al., 2021), our study found that HIBD could significantly reduce BDNF protein expression. Over recent years, some studies have revealed that DEX exerts neuroprotective effects by activating BDNF signaling in other models of neurological impairment, such as the neurotoxicity in developing rats induced by sevoflurane (Dong et al., 2020), cerebral ischemia/reperfusion injury in rats (Li et al., 2018) and kainic acid-induced neural excitotoxicity (Chiu et al., 2019). In the present study, we found that DEX treatment significantly enhanced the expression of BDNF in hypoxic-ischemic neonatal rats. It is well known (Zhou et al., 2021) that BDNF is widely expressed in all forms of brain cells and is particularly highly expressed in M2 microglia. Therefore, we chose to detect BDNF expression in microglia. In our study, we found that BDNF was expressed in microglia by performing double immunofluorescence staining; the immunofluorescence results were the same as those obtained from western blotting. Collectively, our results indicated that the effect of DEX in neonatal rats following HIBD was associated with BDNF. Therefore, our next step was to investigate how DEX promotes the expression of BDNF.

Neuroinflammation is one of the major causes of injury in neonatal HIBD and can induce significant mortality in neuronal cells and other types of brain cells (Millar et al., 2017). Strong evidence now indicates that chronic inflammation, as determined by increasing levels of key soluble pro-inflammatory cytokines such as tumor necrosis factor -α (TNF-α), interleukin-1β (IL-1β) and interleukin-6 (IL-6), plays an important role in the development of various neurological disorders, including neonatal HIBD (Li et al., 2020; Khodarahmi et al., 2021; Yu et al., 2021). In the present study, we determined the expression levels of TNF-α, IL-1β and IL-6 proteins by western blotting; our results concurred with previous researchers (Aly et al., 2006; Yang et al., 2016; Borjini et al., 2019) in that HIBD induced a robust inflammatory response in the brain. We found that the expression levels of TNF-α, IL-1β and IL-6 were significantly higher in the HI group than in the sham group. Several types of brain injuries have been reported to be relieved by the anti-inflammatory properties of DEX (Qiu et al., 2020). Similarly, we also found that DEX treatment significantly reduced the expression levels of TNF-α, IL-1β and IL-6 proteins in hypoxic-ischemic neonatal rats. The microglia are the resident macrophages of the central nervous system and are the first cell type to become activated after neonatal HIBD (Kaur et al., 2007). Activated microglia can migrate to the injured region of the brain and cause secretion of inflammatory cytokines, nitric oxide, free radicals and glutamate (Kaur and Ling, 2009). These secretions can aggravate the inflammatory response. It was previously reported that individual drugs could protect the neonatal brain following HIBD by blocking microglial activation (Dommergues et al., 2003). Our immunofluorescence staining also found demonstrated that DEX treatment significantly blocked activation of the microglia by immunofluorescence staining. Based on the fact that BDNF is widely expressed in all kinds of brain cells (Zhou et al., 2021), we therefore hypothesized that DEX promoted the survival of neurons and other brain cells by inhibiting microglial activation and by reducing inflammation to enhance the expression of BDNF.

There are two phenotypes for microglia: the classic phenotype M1 and the alternative phenotype M2. M1 microglia aggravate brain injury and impede the repair of the central nervous system by producing pro-inflammatory factors such as IL-6 and IL1-β. In contrast, M2 microglia promote the repair of neural damage and the survival of brain cells by releasing anti-inflammatory factors, such as IL-10, IL-4 and some neurotrophic factors, including BDNF (Hu et al., 2012). Our study is the first to demonstrate that DEX promotes M1 to M2 conversion in the microglia of neonatal rats following HIBD. This finding is similar to that of a previous study that was verified by *in vitro* experiments (Degos et al., 2013). Therefore, modulation of M1/M2 polarization in the microglia may become a potent mechanism by which DEX exerts anti-inflammatory effects and enhances the expression of BDNF in hypoxic-ischemic neonatal rats.

This study has some limitations that need to be considered. First, the dose of DEX used in this study was consistent with previous research involving neuroprotection. Future studies should evaluate additional doses to investigate the dose-effect relationship and select an optimal dose that promotes neurogenesis in the SVZ to improve the olfactory cognitive dysfunction in neonatal HIBD. Second, little is known about the BDNF signaling pathway which plays a crucial role in neurogenesis. In our future research, we will investigate the association between the BDNF\TrkB signaling pathway, neonatal HIBD and DEX. In addition, we will use specific inhibitors to demonstrate that the BDNF\TrkB signaling pathway may play a key role in the beneficial effects of DEX treatment. Third, we specifically focused on the effect of DEX on neurogenesis in the SVZ of neonatal rats with HIBD. It is not yet known whether DEX can promote neurogenesis in the SGZ of neonatal rats with HIBD. Finally, the ability of DEX to regulate the polarization of microglia was determined only by semi-quantification of double immunofluorescence staining; other experimental techniques, such as western blotting and PCR, are now needed to better evaluate the effect of DEX in the polarization of microglia. In addition, we aim to investigate the specific internal mechanisms involved in our future study.

## 5 Conclusion

In conclusion, our study demonstrated that HIBD can cause severe olfactory cognitive dysfunction in neonatal rats by disrupting neurogenesis in the SVZ. Furthermore, our results showed that DEX treatment improved olfactory cognitive dysfunction in hypoxic-ischemic neonatal rats by promoting neurogenesis in the SVZ and enhancing the expression of BDNF in microglia; these events were associated with the modulation of M1/M2 polarization in the microglia and anti-inflammatory effects. Our findings suggest that olfactory cognitive dysfunction may be the primary presenting symptom of neurological sequelae in neonatal HIBD and that promoting neurogenesis in the SVZ could be an effective and promising candidate target to confer neuroprotection against the neurological sequelae of neonatal HIBD. However, we consider our results preliminary at this stage. We hope that this study may lead to further research on this topic by providing a new target, direction, and a theoretical basis for the treatment of various neurological dysfunctions in neonates caused by HIBD.

## Data Availability

The original contributions presented in the study are included in the article/Supplementary Material, further inquiries can be directed to the corresponding author.
